# Evaluation of Oral Function Development and Eating Habits in Children Aged 5–7 Years

**DOI:** 10.1111/joor.70213

**Published:** 2026-05-15

**Authors:** Jun Kawamura, Susumu Kanno, Teppei Shintani, Misaki Matsui, Yuriko Komagamine, Hitomi Soeda, Anna Miyayasu, Maiko Iwaki, Shunsuke Minakuchi, Yohei Hama, Kazuto Okabayashi, Manabu Kanazawa

**Affiliations:** ^1^ Central Research Laboratory Lotte Co., Ltd. Tokyo Japan; ^2^ Department of Gerodontology and Oral Rehabilitation, Division of Gerontology and Gerodontology, Graduate School of Medical and Dental Sciences Institute of Science Tokyo Tokyo Japan; ^3^ Department of Digital Dentistry Institute of Science Tokyo Tokyo Japan

**Keywords:** child development, eating habits, feeding and eating disorders of childhood, mastication, nutrition education, oral health

## Abstract

**Background:**

The number of children with poor oral function, known as developmental insufficiency of oral function (DIOF), has increased, negatively affecting children's growth and development. Research on the overall prevalence of DIOF in children is limited.

**Objective:**

We aimed to investigate the prevalence of DIOF in community‐dwelling children aged 5–7 years and examine its relationship with eating habits that may affect oral function.

**Methods:**

We analysed data from children who participated in the Survey on All‐Generation Masticatory Strength and Eating Habits. Chi‐square tests were used to examine the relationship between the incidence of DIOF and participant characteristics. Logistic regression was used to explore the relationship between eating habits and participant characteristics, namely oral function and DIOF incidence.

**Results:**

Ninety‐two children (aged 5.6 ± 0.1, 47 males) were included in the final analysis, 23% of whom had DIOF. Children who preferred hard foods were less likely to have DIOF (adjusted odds ratio = 0.636, 95% confidence interval 0.405–0.998, *p* = 0.049). The frequency of hard food intake had no influence on DIOF incidence.

**Conclusion:**

Approximately one in four children who participated in the study had DIOF. A preference for hard food was associated with a reduced risk of DIOF, indicating the importance of dietary texture in oral function development. Given the cross‐sectional design of this study, further research is needed to explore the development of oral function through oral and dietary education.

## Graphical abstract

Summary of ‘Evaluation of Oral Function Development and Eating Habits Among Children Aged 5 to 7 Years’.

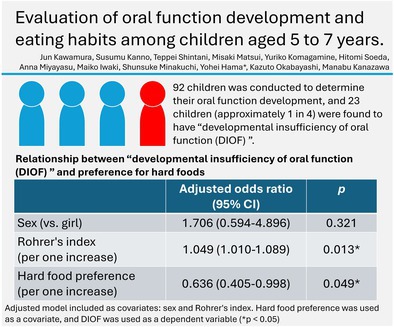

## 1 Introduction

Recently, the increasing consumption of soft foods and other lifestyle factors have raised concerns regarding the poor development of oral function in children [1]. Oral functions include eating (mastication), swallowing and speaking, with most children acquiring these essential functions by 6 and 7 years of age. As poor oral function development can affect nutritional status and physical and social growth [2, 3], studies have been conducted to evaluate methods for assessing oral function in children [4].

In Japan, a condition termed developmental insufficiency of oral function (DIOF) was introduced in 2018. This condition is characterized by underdeveloped or abnormal oral functions, including eating and speaking, requiring specialized support owing to individual or environmental factors [5]. However, following its recent classification, DIOF‐related reports [6] and research on individualized preventive methods are lacking. While there have been studies on DIOF in children who attend dental clinics [7, 8], research on community‐dwelling children remains limited [9]. Although previous studies examining developmental disorders among children in nursery schools have revealed that the rate of DIOF is nearly 40%, surveys conducted in dental clinics have reported a prevalence of 2.4% or less than 10%. Thus, information about DIOF, including its prevalence and effective treatment methods, remains limited.

Given that oral function is closely related to dietary status [10], dietary guidance may represent a potential strategy for preventing oral dysfunction in children. In recent years, food education programmes have become increasingly popular in schools, suggesting an avenue for dietary guidance implementation that could also contribute to oral dysfunction prevention. School‐based programmes have been shown to be effective in promoting oral hygiene behaviours among children [11, 12].

To verify this hypothesis, this study aimed to evaluate oral function and DIOF and clarify their association with eating habits in children aged 5–7 years using data from the 2023 Investigative Research on Masticatory Strength and Eating Habits for All Generations, conducted by Lotte Co. Ltd. and the Institute of Science Tokyo (formerly Tokyo Medical and Dental University).

## 2 Methods

### 2.1 Study Setting and Participants

We obtained data from children aged 5–7 years who participated in the 2023 Investigative Research on Masticatory Strength and Eating Habits for All Generations, which examined the state of oral function across the following age groups: 5–7 years (*n* = 92), 20–39 years (*n* = 194), 40–64 years (*n* = 198), 65–74 years (*n* = 199) and ≥ 75 years (*n* = 195). The trial was registered in the University Hospital Medical Information Network clinical trial registration system (ID: UMIN000050652). Children within the specified age group (5–7 years), regardless of sex, were included in the study. The exclusion criteria were: (i) difficulty chewing gum because of temporomandibular joint disorder, food allergy, or similar conditions; and (ii) cognitive impairments affecting study participation.

All children were accompanied by their parents, who provided written informed consent on their behalf. Informed assent was also obtained from the children themselves. All procedures were explained to participants in advance using a consent form approved by the Ethics Committee of the Faculty of Dentistry at the Institute of Science Tokyo (approval no. D2022‐80). The study was conducted in accordance with the principles of the Declaration of Helsinki. Ethical considerations, including handling of personal information, obtaining informed consent, participant burdens, risks and benefits, conflicts of interest and information disclosure, followed our institutional regulations.

### 2.2 Measurements

#### 2.2.1 Oral Function

The following oral functions were assessed: masticatory function [13], maximum occlusal force [14], maximum tongue pressure [15] and dental status.

Masticatory function was evaluated using a colour‐changing chewing gum (3.0 g, xylitol mastication check gum; Lotte Co. Ltd., Tokyo, Japan), the colour of which changes from green to red during chewing. Each participant chewed the gum 60 times at a rate of 1 chew per second, not limited to left and right chewing measurements. After chewing, the gum was placed in a transparent bag and smoothed to a thickness of 1.5 mm using two glass plates. Measurements were taken at five locations using a colorimeter (CR13, Konica Minolta, Tokyo, Japan), ΔE values were calculated from each colour space coordinate value (L*, a*, b*), and the average was obtained. The baseline values for L*, a* and b* before mastication were 72.3, −14.9 and 33, respectively.

$$∆E=\sqrt{{\left(L*-72.3\right)}^{2}+{\left(a*-\left(-14.9\right)\right)}^{2}+{\left(b*-33.0\right)}^{2}}$$

The maximum occlusal force was measured using an oral function monitor (Oramo‐bf; Sumitomo Riko Co. Ltd., Aichi, Japan). A sensor sheet was placed at the tip of the Oramo‐bf machine between the maxillary and mandibular dental arches, and participants were instructed to clench with maximum force for 3 s. The maximum value was then used in the analysis.

Tongue pressure was measured using a tongue pressure‐measuring instrument (TPM‐01; GC Corporate Center, Tokyo, Japan). The participants were instructed to place the balloon sensor in front of their palate and press against it using their tongue for 3 s. Measurements were performed thrice, and the maximum value was used as the representative value.

#### 2.2.2 DIOF

DIOF [5] was assessed by dentists using a checklist for dental diagnostics developed in 2020 (Table 1). While both pre‐ and post‐weaning questionnaires exist, the post‐weaning questionnaire was used in this study. The participants (or their parents) completed a self‐administered questionnaire. All dentists received training in advance, and the assessment criteria were standardized to ensure diagnostic uniformity.

**TABLE 1:** Checklist for DIOF after weaning (excerpt) and the number of affected participants (duplicates included).

Function	Classification	Item	Overall (*n* = 92)	DIOF		*p* ^a^
				Applicant (*n* = 21)	Non‐applicant (*n* = 71)	
Eating	Chewing function	Delayed tooth eruption	4 (4%)	4 (19%)	0 (0%)	< 0.001*
		Abnormalities in dentition and occlusion due to functional factors	1 (1%)	1 (5%)	0 (0%)	0.064
		Have caries that affect chewing	1 (1%)	1 (5%)	0 (0%)	0.064
		Cannot bite down with force	6 (7%)	4 (19%)	2 (3%)	0.008*
		Chewing time is too long or too short	16 (17%)	14 (67%)	2 (3%)	< 0.001*
		Have an irregular chewing pattern	9 (10%)	7 (33%)	2 (3%)	< 0.001*
	Swallowing function	Tongue protrusion (residual infantile swallowing) is present (post‐weaning)	6 (7%)	4 (19%)	2 (3%)	0.008*
	Eating behaviour	Inconsistent feeding amount, amount of food consumed, frequency	33 (36%)	12 (57%)	21 (30%)	0.021*
Speaking	Articulation function	Speech problems (substitutions, omissions, distortions)	13 (14%)	5 (24%)	8 (11%)	0.147
		Incomplete lip closure (lips cannot be closed when at rest)	38 (41%)	13 (62%)	25 (35%)	0.029*
		Have oral habits	23 (25%)	7 (33%)	16 (23%)	0.315
		Abnormal lingual frenulum	4 (4%)	1 (5%)	3 (4%)	0.916
Other functions	Nutrition (physique)	Being thin or obese (evaluated by Kaup's index for those < 6 years old and by Rohrer's index for those ≥ 6 years old)	4 (4%)	2 (10%)	2 (3%)	0.186
	Others	Mouth breathing present	36 (39%)	11 (52%)	25 (35%)	0.157
		Snoring during sleep	18 (20%)	7 (33%)	11 (15%)	0.070

*Note:* Data are shown as the number of participants.

Abbreviation: DIOF, developmental insufficiency of oral function.

^a^ Differences in variables among those with and without DIOF were analysed using the *χ* ^2^‐test for categorical variables (*p < 0.05).

For the ‘being thin or obese’ category, we followed the established definitions of DIOF and used the Kaup index for children aged < 6 years and the Rohrer index for children aged ≥ 6 years. Self‐reported data were used for height and weight. For the Kaup index, a score of ≥ 22 was defined as obese and a score of < 13 was defined as underweight. For the Rohrer index, a score of ≥ 160 was defined as obese and a score of < 100 was defined as underweight. The calculation methods for each index are as follows:

Kaup index=weightgheightcm2×10

Rohrer index=weightgheightcm3×104

regarding DIOF classification, children were classified as having DIOF based on the official DIOF criteria, meeting ≥ 2 items in the Eating and Speaking function categories, with ≥ 1 item in the Chewing function category within the Eating function category.

#### 2.2.3 Nutrient Intake

Nutrient intake was assessed using the brief‐type self‐administered diet history questionnaire 15Y (BDHQ‐15Y) [16, 17].

#### 2.2.4 Eating Habits

Eating habits were assessed using a 5‐point scale that evaluated the frequency of hard food intake (‘always’, ‘quite a bit’, ‘occasionally’, ‘hardly’ and ‘never’) and hard food preference (‘like quite a lot’, ‘like quite a bit’, ‘neither’, ‘do not like very much’ and ‘do not like at all’). Following previous research, several hard foods were used as examples, including rice crackers, walnuts and carrot sticks [18]. Questions that were difficult for children to answer were answered by the parents.

#### 2.2.5 Covariates

The body mass index (BMI) and Rohrer's index of participants were assessed using self‐reported height and weight data as covariates [19]. Age and sex were also self‐reported and used as covariates.

### 2.3 Statistical Analysis

Statistical significance was set at *p* < 0.05. All statistical analyses were conducted using IBM Statistical Package for Social Sciences for Windows, version 28.0 (IBM Corporation, Armonk, NY, USA). The Mann–Whitney U and *χ* ^2^ tests were used to compare oral function, eating habits and DIOF indicators between children with and without DIOF. A binary logistic regression model was used to calculate odds ratio (OR) and 95% confidence interval (CI) for each association, with the prevalence of DIOF as the dependent variable and eating habits, sex and the Rohrer's index as the independent variables. Sex and the Rohrer's index were used as covariates to account for differences in growth between the sexes and for differences in body size.

## 3 Results

This study included 92 participants, 21 of whom were diagnosed with DIOF (approximately 23%). The chief complaints and corresponding dentist evaluations are shown in Table 1. The most frequently reported indicators of DIOF were ‘incomplete lip closure (lips cannot be closed when at rest)’ in the overall study population and ‘chewing time is too long or too short’ among those diagnosed with DIOF. In particular, these two indicators were present in over 60% of the DIOF group and were significantly more common than in the non‐DIOF group (*p* = 0.029 and *p* < 0.001, respectively). Other common indicators included ‘delayed tooth eruption’, ‘cannot bite down with force’, ‘have an irregular chewing pattern’, ‘tongue protrusion (residual infantile swallowing) is present (post‐weaning)’ and ‘inconsistent feeding amount, amount of food consumed, frequency’.

Table 2 shows the comparison of basic participant characteristics, oral function, nutrient intake and other items between participants with and without DIOF. Participants without DIOF had significantly higher nutrient intake (energy: *p* = 0.002, protein: *p* < 0.001, fat: *p* = 0.003, carbohydrate: *p* = 0.025, calcium: *p* < 0.001, iron: *p* = 0.003, vitamin A: *p* < 0.001, vitamin D: *p* = 0.015, vitamin B_1_: *p* = 0.012, vitamin B_2_: *p* < 0.001, vitamin C: *p* = 0.049) than those with DIOF.

**TABLE 2:** Comparison of oral function and other items between participants with and those without DIOF.

		Overall (*n* = 92)	DIOF		*p* ^a^
			With (*n* = 21)	Without (*n* = 71)	
Basic attributes	Age, years	6 (5–6)	6 (5–6)	6 (5–6)	0.793
	Sex (male), *n*	47 (51.1%)	9 (42.9%)	38 (53.5%)	0.461
	Height, cm	112 (108–117)	110 (105–116)	113 (108–118)	0.228
	Body weight, kg	19.2 (17.4–20.8)	19.0 (16.8–20.9)	19.3 (17.5–20.8)	0.834
	Body mass index, kg/m^2^	15.1 (14.1–15.9)	15.4 (14.4–17.0)	15.0 (14.1–15.8)	0.113
Oral function	Number of permanent teeth, *n*	1 (0–4)	1 (0–6)	1 (0–4)	0.758
	Number of baby teeth, *n*	19 (17–20)	19 (15–20)	20 (17–20)	0.369
	Maximum occlusal force, N	343 (239–442)	301 (226–440)	355 (250–443)	0.329
	Maximum tongue pressure, kPa	19.5 (14.3–28.7)	19.9 (17.7–28.8)	19.4 (13.7–28.6)	0.573
	Masticatory function (ΔE), score	35.9 (28.5–43.3)	35.8 (31.2–45.0)	35.9 (27.9–42.0)	0.415
Nutritional status	Energy, kcal	2051 (1707–2503)	1683 (1504–2157)	2111 (1803–2551)	0.002*
	Protein, g	71.0 (57.0–84.5)	53.6 (42.2–67.7)	73.0 (62.2–86.8)	< 0.001*
	Fat, g	79.4 (64.0–99.2)	66.0 (40.2–82.8)	81.0 (67.0–100)	0.003*
	Carbohydrate, g	250 (223–308)	223 (188–299)	259 (229–309)	0.025*
	Calcium, mg	746 (570–991)	569 (358–770)	848 (655–1092)	< 0.001*
	Iron, mg	7.27 (5.67–8.62)	5.58 (4.19–7.46)	7.74 (5.83–8.70)	0.003*
	Vitamin A, μg	363 (262–497)	257 (174–346)	399 (304–532)	< 0.001*
	Vitamin D, μg	7.79 (4.35–10.57)	5.06 (2.98–8.51)	8.88 (4.63–11.0)	0.015*
	Vitamin B_1_, mg	0.79 (0.64–0.93)	0.64 (0.49–0.87)	0.80 (0.68–0.98)	0.012*
	Vitamin B_2_, mg	1.58 (1.27–1.94)	1.11 (0.81–1.57)	1.67 (1.33–1.97)	< 0.001*
	Vitamin C, mg	96.7 (71.4–132.5)	71.6 (50.7–126.6)	99.0 (75.4–133.5)	0.049*

*Note:* Data are shown as the median (interquartile range) for quantitative measures and as the number of participants (percentages) for all qualitative measures.

Abbreviation: DIOF, developmental insufficiency of oral function.

^a^ Differences in variables among those with and without DIOF were analysed using the *χ* ^2^‐test for categorical variables and Mann–Whitney *U*‐test for continuous variables (*p < 0.05).

The binomial logistic regression analysis revealed that a preference for hard food was associated with a reduced risk of DIOF (Table 3: adjusted OR [aOR] = 0.636, 95% CI 0.405–0.998, *p* = 0.049). However, there was no correlation between the frequency of hard food intake and the prevalence of DIOF (aOR = 0.670, 95% CI 0.411–1.093, *p* = 0.109). Table 4 shows the frequency of hard food intake and hard food preference.

**TABLE 3:** Binary logistic regression analysis of DIOF and hard food intake frequency and preference.

		Crude odds ratio (95% CI)	*p* ^a^	Adjusted odds ratio (95% CI)	*p* ^b^
DIOF	Frequency of hard food intake	0.737 (0.463–0.174)	0.199	0.670 (0.411–1.093)	0.109
	Hard food preference	0.674 (0.438–1.037)	0.072	0.636 (0.405–0.998)	0.049*

*Note:* Data are shown with 95% CI.

Abbreviations: CI, confidence interval; DIOF, developmental insufficiency of oral function.

^a^ Results of binomial logistic regression analysis when frequency of hard food intake or hard food preference was used as a covariate, and DIOF was used as a dependent variable.

^b^ Adjusted model included sex and the Rohrer's index. The frequency of hard food intake or preference was used as a covariate, and DIOF was used as a dependent variable (*p < 0.05).

**TABLE 4:** Table of frequency of intake and preference of hard foods.

Frequency of hard food intake	Hard food preference					Total
	Like quite a lot	Like quite a bit	Neither	Do not like very much	Do not like at all	
Always	3	3	0	0	0	6
Quite a bit	8	14	2	0	0	24
Occasionally	0	16	14	3	0	33
Hardly	1	3	3	11	2	20
Never	0	1	2	1	5	9
Total	12	37	21	15	7	92

## 4 Discussion

In this study, we evaluated the oral function of 92 children aged 5–7 years. Among them, 21 had DIOF. Significant differences were observed across various indicators, including nutritional status, between children with and those without DIOF. In a previous study of 329 patients aged ≤ 15 years [7], 32 (9.7%) patients who visited a specialist outpatient clinic for eating and swallowing problems were diagnosed with DIOF. In addition, a study using the National Database reported a DIOF prevalence of 2.4% among patients attending a dental clinic [8]. In contrast, the prevalence of DIOF in the current study was considerably high, at approximately 23%, and another study involving nursery school children reported a prevalence rate of approximately 40% [9]. There are two possible reasons for the high prevalence of DIOF in this study. First, this study did not target patients who attend a dental clinic but instead analysed data of community‐dwelling children, including those who do not attend a dental clinic. Similarly, a previous study including children who did not attend a dental clinic revealed a high prevalence rate of 40% [9]. This discrepancy in results may be due to differences in the study populations. These findings suggest that the prevalence of DIOF is generally high among children but that it may not lead to dental visits, potentially due to low parental awareness of DIOF, leading to lower rates of oral function developmental disorder in clinical settings compared to the results of this study and studies targeting preschool children. Therefore, it is important to increase awareness of DIOF and promote healthy oral function development in children.

Second, the increased prevalence of DIOF in the present study could be attributed to the younger target age group of our participants, who were at or below elementary school age. Considering that social development improves substantially upon entering elementary school, social interactions at this stage of growth may have a major effect on oral function. Currently, tongue pressure and lip closure strength are used as reference assessment items for oral dysfunction. However, given that these values are known to increase with age, differences have been reported, particularly before and after entering elementary school [20, 21, 22]. Since the present study targeted students entering elementary school, the difference observed may reflect a transitional period in oral function development. Previous studies reporting a low prevalence of DIOF targeted participants with a wide range of ages, including junior high and high school students [7, 8]. If the prevalence of DIOF is low in older children, it is possible that the prevalence rate would be high in our study and studies targeting nursery schools [9]. Similarly, previous studies targeting junior high and high school students would also show low prevalence rates [7, 8]. Future studies should investigate the prevalence of DIOF in elementary, junior high and high school students who do not visit dentists.

In the present study, the most commonly reported indicators of DIOF were incomplete lip closure and chewing time. Although poor bite alignment due to dental problems may contribute to these abnormalities, their frequency exceeded the number of reported dental problems, suggesting underdevelopment of perioral muscles. Previous studies have proposed oral function training methods such as chewing bubble gum in children [23] and oral exercises in older individuals [24]. Our findings highlight the need for further research on oral function training in children.

Upon comparing oral function between children with and without DIOF, no significant differences were found. This may be attributed to the small number of children with DIOF in this study. Additionally, the nutrient intake of children with DIOF was significantly lower than that of those without DIOF. Although nutrient intake was within the recommended amount for their age [25], the dietary assessment tool used in this study (BDHQ15Y) surveys weekly food intake frequency and calculates nutrient intake. Although it is adjusted for children, it is intended for children aged 6–15 years. Therefore, the calculated intake may have been higher than expected due to the age adjustments made, the validity of which remains under review. If this was the case, participants with DIOF may have had reduced nutrient intakes. Nonetheless, this finding suggests that DIOF may make it difficult to eat, leading to reduced intake, and that DIOF may result from the reduced chewing frequency when small amounts of food are consumed.

In this study, we also examined the relationship between DIOF and children's eating habits, namely the frequency of hard food intake and preference for hard food. The question about the frequency of hard food intake aimed to determine whether eating hard food strengthens the muscles around the mouth and promotes the development of oral function. The question about the preference for hard food was included because, although parents typically prepare meals for their children, children may select their own portions from shared dishes during family meals. In such cases, the preference survey also considered the possibility that children may eat less of these foods than their parents are aware of due to their preference. As shown in Table 4, there was in fact a certain degree of correlation between preference for hard foods and frequency of intake, and there was a subset of participants who liked hard foods but did not eat them often. Conversely, there were no participants who disliked hard foods but ate them often. The binomial logistic regression analysis adjusted for age, sex and the Rohrer's index revealed a correlation between DIOF and preference for hard food. Although no association was observed between the frequency of hard food intake and DIOF, the responses to this item were somewhat concentrated in the intermediate categories. Such response distributions may reduce the ability to detect associations; therefore, this finding should be interpreted with caution. While the cross‐sectional design of this study limits causal interpretation, it is possible that a preference for hard foods may influence DIOF prevention or that developing DIOF may lead to a tendency to dislike hard foods. To the best of our knowledge, there has been no previous research on the relationship between children's eating habits and oral function development. Nonetheless, the relationship between oral function and obesity in children has been clearly established [26, 27], suggesting a correlation between oral function and eating habits. Various studies on oral function and nutritional intake in older individuals have been conducted, for example, where declining oral function leads to avoidance of hard food consumption, which leads to a further deterioration [28, 29, 30]. Therefore, eating habits and nutritional guidance may be important for promoting oral function development in children. Previous research has suggested that parental communication alone is insufficient for promoting oral hygiene [11]. However, the importance of oral hygiene programmes implemented through schools is considered effective for changing behaviours among children [12]. Therefore, we believe creating opportunities for children to learn about the importance of eating hard foods and developing mouth muscles for chewing in an educational setting is critical.

To the best of our knowledge, this is the first study to investigate oral function and eating habits among children entering elementary school in a local community. However, this study had some limitations. First, the cross‐sectional study design means that a clear causal relationship could not be established. Second, responses may lack accuracy given the age of the participants. While parents answered the more difficult questions on behalf of their children, inaccuracies may still exist. Third, there may be some aspects of diet that parents are unaware of. As this study was conducted during summer vacation, school lunches and other factors are unlikely to have influenced the BDHQ results; however, in everyday life, these may contribute to nutrient intake not reflected in this study. Fourth, this study used a checklist‐based assessment of DIOF that is currently applied in routine paediatric dental practice in Japan. Although this checklist reflects real‐world clinical evaluation, some items lack strictly quantitative criteria and may involve subjective judgement. Moreover, several items rely on parental reports, which may introduce potential measurement bias. Therefore, the possibility of bias associated with this assessment tool cannot be completely excluded. Future studies are needed to further evaluate the validity and reliability of this checklist‐based assessment. Lastly, the study may have omitted other important factors related to DIOF, such as dental history and dental behaviour, warranting further investigation.

In conclusion, approximately 23% of children aged 5–7 years were found to have DIOF. The most common indicators were incomplete lip closure and chewing time, which are thought to be influenced by perioral muscle underdevelopment. Our findings indicated a potential link between oral function development and eating habits. Therefore, dietary guidance for children may be important for supporting the development of oral function. Given its potential effect on overall growth, this study may serve as a basis for future research on promoting oral function development in children, investigating the prevalence of DIOF in children of elementary school age and older who do not attend dental clinics and informing intervention studies aimed at improving DIOF through dietary guidance.

## Funding

This work was supported by Lotte Co., Ltd., 23‐009‐E.

## Conflicts of Interest

J. K., S. K., T. S., M. M. and K. O. are employees of Lotte Corporation. Y. K., S. M., Y. H. and M. K. are affiliated with the Institute of Science Tokyo and have collaborated with Lotte Corporation on this research and have received research funding.

## Data Availability

Research data are not shared.
